# Virtual monochromatic images for coronary artery imaging with a spectral photon-counting CT in comparison to dual-layer CT systems: a phantom and a preliminary human study

**DOI:** 10.1007/s00330-023-09529-9

**Published:** 2023-03-15

**Authors:** Joel Greffier, Salim A. Si-Mohamed, Hugo Lacombe, Joey Labour, Djamel Djabli, Sara Boccalini, Mohammad Varasteh, Marjorie Villien, Yoad Yagil, Klaus Erhard, Loic Boussel, Jean-Paul Beregi, Philippe C. Douek

**Affiliations:** 1 IMAGINE UR UM 103, Montpellier University, Department of Medical Imaging, Nîmes University Hospital, Nîmes, France; 2 University Lyon, INSA-Lyon, University Claude Bernard Lyon 1, UJM-Saint Etienne, CNRS, Inserm, CREATIS UMR 5220, U1206, F‐69621, 7 Avenue Jean Capelle O, 69100 Villeurbanne, France; 3 Department of Cardiothoracic Radiology, Louis Pradel Hospital, Hospices Civils de Lyon, 59 Boulevard Pinel, 69500 Bron, France; 4 Philips Healthcare, Suresnes, France; 5 Philips Healthcare, Haifa, Israel; 6 Philips Healthcare, Hamburg, Germany; 7 Department of Radiology, Croix Rousse Hospital, Hospices Civils de Lyon, 103 Gd Rue de la Croix-Rousse, 69004 Lyon, France

**Keywords:** Multidetector computed tomography, Image enhancement, Radiography, dual-energy scanned projection, Diagnosis, Coronary vessels

## Abstract

**Objectives:**

To evaluate the quality of virtual monochromatic images (VMIs) from spectral photon-counting CT (SPCCT) and two energy-integrating detector dual-energy CT (EID-DECT) scanners from the same manufacturer, for the coronary lumen.

**Methods:**

A 21-cm section of the Mercury v4.0 phantom was scanned using a cardiac CT protocol. VMIs from 40 to 90 keV were reconstructed using high-resolution (HR) parameters for EID-DECT and SPCCT (CB and HRB kernels at 0.67 mm slice thickness, respectively). Ultra-high-resolution (UHR) parameters were used in addition to SPCCT (detailed-2 kernel, 0.43 mm slice thickness). Noise-power-spectrum (NPS), task-based transfer function (TTF), and detectability index (*d′*) were computed for 2-mm-diameter lumen detection. In consensus, two radiologists analyzed the quality of the images from 8 patients who underwent coronary CTA on both CT systems.

**Results:**

For all keV images, *f*_peak_, *f*_50_, and *d′* were higher with SPCCT. The *f*_peak_ and *f*_50_ were higher with UHR-SPCCT with greater noise and lower *d′* compared to those of the HR-SPCCT images. Noise magnitude was constant for all energy levels (keV) with both systems, and lower with HR images, and *d′* decreased as keV decreased. Subjective analysis showed greater lumen sharpness and overall quality for HR and UHR-SPCCT images using all keV, with a greater difference at low keV compared to HR-EID-DECT images.

**Conclusion:**

HR and UHR-SPCCT images gave greater detectability of the coronary lumen for 40 to 90 keV VMIs compared to two EID-DECT systems, with benefits of higher lumen sharpness and overall quality.

**Key Points:**

*• Compared with 2 dual-energy CT systems, spectral photon-counting CT (SPCCT) improved spatial resolution, noise texture, noise magnitude, and detectability of the coronary lumen.*

*• Use of ultra-high-resolution parameters with SPCCT improved spatial resolution and noise texture and provided high detectability of the coronary lumen, despite an increase in noise magnitude.*

*• In eight patients, radiologists found greater overall image quality with SPCCT for all virtual monochromatic images with a greater difference at low keV, compared with dual-energy CT systems.*

**Supplementary Information:**

The online version contains supplementary material available at 10.1007/s00330-023-09529-9.

## Introduction

Virtual monochromatic images (VMIs) are a key feature of spectral CT imaging [1–3]. Their principle relies on simulating tissue attenuation at a particular energy by means of photoelectric and Compton effects. At low energy levels, VMIs provide strong contrast for images of certain tissues (particularly iodine-enhanced images) and VMIs at high energy levels can reduce beam hardening and blooming artifacts [3]. For coronary artery disease imaging, VMIs at 40 to 90 keV are commonly used, both at low- and high-energy levels, depending on the clinical task. For example, the use of low-energy levels of VMIs for coronary CT angiography allows a significant reduction in iodine load [4–6] and improves the quality of coronary plaque components [7], while the use of high-energy levels of VMI leads to quality improvement of the coronary lumen in the presence of a stent [8, 9]. Despite these promising results, the performance of VMIs may be impaired due to the intrinsic limitations of chain detection in current dual-energy CT systems which relies on energy-integrating detectors (EIDs) [10–12].

A new device, the spectral photon-counting CT system (SPCCT), has recently been developed and implemented in high-count-rate CT systems, with clinical capabilities for human imaging [1, 13]. The detection chain relies on new detectors with energy-resolving capabilities called photon-counting detectors (PCDs) [14, 15]. Compared to energy-integrating detectors, photon-counting detectors provide higher spatial resolution, suppress electronic noise, improve contrast-to-noise ratios, and lead to higher dose efficiency [1, 13, 15–24]. The ability to count each photon’s energy also improves sampling of the X-ray spectrum in multiple energy bins. It can discriminate photoelectric and Compton attenuation coefficients more accurately, for better-quality VMIs. A few studies have pointed out higher-quality VMIs obtained with SPCCT [19, 25–28]. However, no studies have ever evaluated the additional value of VMIs using a task-based image quality method to thoroughly characterize noise magnitude and frequency by means of the noise power spectrum (NPS) and the spatial resolution adapted to a specific contrast using a task-based transfer function (TTF) [29–31]. These parameters also allow assessment of the detectability index (*d′*), a mathematical model observer representing the radiologist’s ability to perform a task [30–34]. Altogether, these metrics are often used in addition to, or upstream from, a subjective analysis carried out by radiologists on images from patients or anthropomorphic phantoms [35].

Compared to the 4-cm collimation dual-layer energy-integrating detectors of dual-energy CT in phantom studies [34, 36], conventional SPCCT images have outperformed detectability in coronary imaging tasks, and in human studies [18, 36]. Yet it is still unknown whether VMIs may benefit from the improved technical aspects of SPCCT technology for coronary artery disease imaging. This study evaluated the quality of VMIs from a clinical prototype spectral photon-counting CT (SPCCT) scanner and two dual-energy CT scanners with dual-layer energy-integrating detectors (EID-DECT) for coronary lumen imaging, from 40 to 90 keV, using a task-based image quality assessment.

## Materials and methods

### Phantoms

A 21-cm-diameter section of the Mercury v4.0 phantom (Gammex) (Supplemental Fig. 1) was used to perform the task-based image quality assessment. The NPS was computed on the homogeneous module (polyethylene background material) (Supplemental Fig. 1.A) and the TTF on the iodine insert at 10 mg/mL (Supplemental Fig. 1.B).

### CT systems

The SPCCT system (Philips Healthcare) is a large-field-of-view (50 cm in-plane) clinical prototype CT device equipped with energy-sensitive photon-counting detectors. Pixel pitches are 275 × 275 µm^2^ at the isocenter, bonded to Philips’ proprietary ChromAIX2 application-specific integrated circuit, relying on direct conversion, wide-bandgap semiconductors made of cadmium zinc telluride [37, 38]. Each channel offers pulse-height discrimination with five controllable energy thresholds at 30, 51, 62, 72, and 81 keV for optimal image quality on iodine-enhanced images. Further technical details are available in a previously published study [16].

Dual-layer EID-DECT systems are commercially available platforms offering a dual-energy mode for a 4-cm collimation with IQon CT and an 8-cm collimation with CT7500 (Philips Healthcare).

### Acquisition and reconstruction parameters

Three acquisitions were performed at 120 kVp and 255 mAs using a retrospective ECG-gated helical acquisition at a chosen heart rate (60 bpm) on each CT system. Tube current modulation was disabled. Beam collimation, rotation time, and pitch factor for the EID-DECT scanner were chosen to imitate clinical practice (Table 1).

**Table 1:** Acquisition and reconstruction parameters used on the dual-layer energy-integrating detector dual-energy CT systems (EID-DECT) (IQon CT, CT7500) and spectral photon-counting CT (SPCCT) systems

	Dual-layer EID-DECT 1 (IQon CT)	Dual-layer EID-DECT 2 (CT7500)	SPCCT
Tube voltage (kVp)	120	120	120
Tube current (mAs)	255	255	255
Rotation time (s/rot)	0.27	0.27	0.33
Pitch factor	0.2	0.2	0.318
Collimation	64 × 0.625	128 × 0.625	64 × 0.275
Focal spot (mm × mm)	1.1 × 1.2	1.1 × 1.2	0.6 × 0.7
Displayed CTDI_vol_ (mGy)	25	25	25
iDose^4^ levels	NA	NA	iDose^4^ 6
Spectral	1	1	NA
Reconstruction kernel	CB^‡^	CB^‡^	Detailed 2^†^, HRB^†^
Matrix size (number of pixels)	512 × 512	512 × 512	512 × 512
Field of view (mm)	220	220	220
Slice thickness/increment (mm)	0.67/0.34	0.67/0.34	0.43/0.43 and 0.67/0.34

HRB (high-resolution standard filter) and CB (cardiac standard filter) were used for high-resolution (HR) imaging in combination with 0.67 mm slice thickness. Detailed 2 was used for ultra-high-resolution (UHR) imaging in combination with 0.43 mm slice thickness. Slice thickness was set at 0.43 mm contiguous for the SPCCT, i.e., adapted to the reconstructed in-plane pixel size for isotropic voxel size, and at 0.67 mm for matching the minimal slice thickness available with EID-DECT. Note that slice increment was set at half of the slice thickness for EID-DECT images such as performed in clinical practice. Slice increment was similar for SPCCT images enabled by thinner slice thickness

^†^Detailed 2 and HRB have the same cut-off frequency at 14.6-line pairs, but Detailed 2 filter has higher frequency at 50% and 20% modulation transfer function values

^‡^CB filter cut-off is at 12-line pairs per centimeter

Data were reconstructed during the mid-diastolic phase (78% of the R-R interval) of the cardiac cycle. For EID-DECT, raw data were reconstructed according to clinical practice using high-resolution (HR) parameters, with level 1 of the 7 available levels in the spectral reconstruction algorithm, the highest-frequency kernel available CB (Cardiac Standard) and the minimal slice thickness achievable (0.67 mm), corresponding to HR–EID-DECT images. For SPCCT, raw data were reconstructed with level 6 of an adapted hybrid iterative reconstruction algorithm (iDose^4^). Two different-resolution images were reconstructed with HR-SPCCT images using an HRB kernel and a 0.67-mm slice thickness, and ultra-high-resolution (UHR) SPCCT images were reconstructed using a Detailed-2 kernel and a 0.43-mm slice thickness.

To compare the performance of the SPCCT VMIs with conventional acquisitions, an additional reconstruction was performed with the same parameters. This comparison was not made for EID-DLCT because different reconstruction kernels (CB vs. XCB) and reconstruction algorithms (a spectral reconstruction algorithm vs. the iDose^4^ algorithm) were used for spectral and conventional acquisitions.

For all images and both phantoms, the field of view was set at 220 mm and a standard matrix size of 512 × 512 pixels was used. The other acquisition and reconstruction parameters used for both CT systems are depicted in Table 1.

### Task-based image quality assessment

A task-based image quality assessment was performed using iQMetrix-CT software developed by a working group from the French Society of Medical Physicists (SFPM) [39].

For all CT systems, the NPS was computed on the same volume in the *z*-axis (close to 5.5 mm) using four square regions of interest (ROIs) of 64 × 64 pixels (Supplemental Fig. 1.A). The TTF was computed on the iodine insert at 10 mg/mL, using the circular edge technique [40] on the same volume in the *z*-axis (close to 10.75 mm) (Supplemental Fig. 1.B). To minimize the image-noise effect, the TTF and the NPS were computed from the images of the three acquisitions.

The NPS peak was used to quantify changes in magnitude, and the spatial frequency of the NPS peak (*f*_peak_) was measured to assess noise texture. To quantify the loss/benefit of spatial resolution, the spatial frequency at which the TTF was reduced by 50% (*f*_50_) was measured.

The detectability indexes (*d′*) were computed for one circular clinical task, a lumen of the distal coronary arteries (2 mm in diameter). The contrast between the background material and the iodine insert measured during the TTF calculation, varying with the energy level (keV), was used to compute *d′*.

A non-prewhitening observer model with an eye filter was used. The visual response function proposed by Eckstein et al was used with a 1.5 zoom factor at a viewing distance of 500 mm [41]. The clinical task was represented at a matrix size of 300 pixels and a pixel size of 0.05 mm.

### Subjective image quality

To confirm the results of the phantom study, a preliminary study was then performed on patients. Eight patients undergoing both coronary SPCCT and EID-DECT angiographies between January 2021 and May 2022 were selected to participate in this prospective study. All patients had given written, informed consent, and the study was approved by the institutional review board (Hospices Civils de Lyon, approval number: 2019-A02945-52). Briefly, a coronary CT angiography was performed using a retrospective ECG-gated helical acquisition after injecting an iomeprol bolus (400 mg/mL, Iomeron®; Bracco) at 5 mL/s via a 20-G catheter followed by a saline flush of 20 mL at 4 mL/s, as previously published [36]. The reconstruction parameters were comparable to those of the phantom study. In consensus, two experienced cardiac radiologists (S.S-M., 7 years of experience, and P.D., 30 years of experience) reviewed all images presented in random order on a clinical workstation (Intellispace Portal, Philips Healthcare). They were blinded to the image type, the patient’s identity, the reconstruction kernel, the slice system, and the CT system used. They assessed subjective noise, lumen conspicuity and sharpness, and overall image quality on a qualitative scale ranging from 1 to 5 (1 = unacceptable, 2 = suboptimal, 3 = acceptable, 4 = above average, and 5 = excellent) [36]. A value below 3 was considered as unsatisfactory for clinical use.

### Radiation dose

For all phantom acquisitions, the volume CT dose indexes (CTDI_vol_), determined for a 32-cm-diameter (polymethyl methacrylate) reference phantom, were retrieved from the report available on the CT workstation at the end of all acquisitions. The dose-length-product and CTDI_vol_ were also recorded in the patient study.

## Results

### Noise power spectrum

#### Noise magnitude

For all CT systems, reconstruction kernels, and slice thicknesses, the noise magnitude values were similar according to the energy levels, except at 40 keV for SPCCT (Table 2). For all keVs, the noise magnitude was lower with CT7500 than with IQon CT (−13% ± 0.9%).

**Table 2:** Noise magnitude, spatial frequency of the noise power spectrum peaks (*f*_peak_), and values of task-based transfer function at 50% (*f*_50_) for the iodine insert according to the energy levels (keV) for all CT systems

			40	50	60	70	80	90	Conventional
NOISE magnitude (HU)	SPCCT	Detailed 2—0.43 mm	32.3	30.4	30.0	30.0	30.1	30.2	33.5
		HRB—0.67 mm	18.4	15.2	14.5	14.7	15.0	15.3	16.2
	CT7500	CB—0.67 mm	17.3	16.2	15.6	15.4	15.2	15.1	–
	iQon	CB—0.67 mm	18.4	18.4	17.9	17.7	17.6	17.5	–
*f*_peak_ (mm^−1^)	SPCCT	Detailed 2—0.43 mm	0.04/0.79	0.04/0.79	0.04/0.79	0.04/0.79	0.04/0.79	0.04/0.79	0.04/0.75
		HRB—0.67 mm	0.04/0.26	0.04/0.26	0.04/0.26	0.04/0.26	0.04/0.26	0.04/0.26	0.04/0.34
	CT7500	CB—0.67 mm	0.15	0.15	0.19	0.19	0.19	0.19	–
	iQon	CB—0.67 mm	0.15	0.15	0.15	0.15	0.15	0.15	–
*f*_50_ (mm^−1^)	SPCCT	Detailed 2—0.43 mm	0.59	0.58	0.57	0.56	0.55	0.55	0.71
		HRB—0.67 mm	0.46	0.46	0.47	0.47	0.47	0.47	0.63
	CT7500	CB—0.67 mm	0.32	0.31	0.30	0.29	0.28	0.27	–
	iQon	CB—0.67 mm	0.33	0.33	0.32	0.31	0.30	0.29	–

HRB (high-resolution standard filter) and CB (cardiac standard filter) were used for high-resolution (HR) imaging in combination with 0.67 mm slice thickness. Detailed 2 was used for ultra-high-resolution (UHR) imaging in combination with 0.43 mm slice thickness

On the HR-SPCCT images, the noise magnitude was lower than with IQon CT (−16% ± 2.4%) except at 40 keV, and in the same range as with CT7500 (−2% ± 5.2%). On UHR-SPCCT images, the noise magnitude values were higher than with IQon CT or CT7500. For all VMIs, the noise magnitude was higher on the UHR-SPCCT images than on the HR-SPCCT images with an average increase of 97% ± 11.1%. The noise magnitude was lower for all energy levels than conventional images, except at 40 keV for HR images.

### Noise texture

For SPCCT and each reconstruction kernel, the *f*_peak_ values were similar at all keVs (Table 2). For the IQon CT, the *f*_peak_ was 0.15 mm^−1^ for all energy levels. For CT7500, the *f*_peak_ was 0.15 mm^−1^ at 40 and 50 keV and 0.19 mm^−1^ for the other energy levels.

**Fig. 1:**
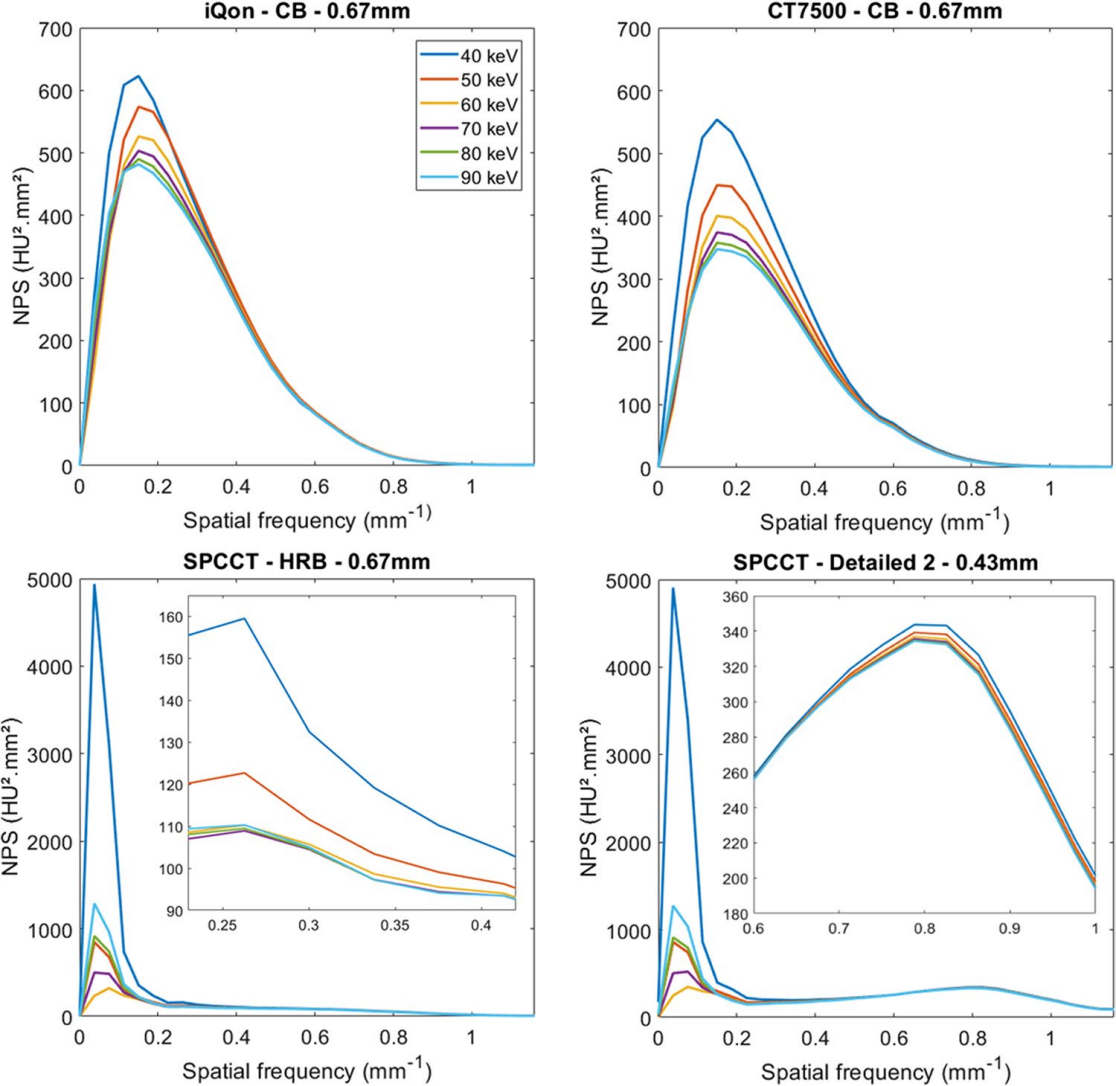
Noise power spectrum curves according to the energy levels obtained for dual-energy images from two dual-layer CT scanners with energy-integrating detectors (IQon CT, CT7500) and a spectral photon-counting CT (SPCCT) scanner. For SPCCT, raw data were reconstructed using high-resolution parameters with HRB kernel and 0.67-mm slice thickness, and ultra-high-resolution parameters with Detailed-2 kernel and 0.43 mm slice thickness

Two peaks on the NPS curves were found on the SPCCT images, one for spatial frequencies around 0.04 mm^−1^ that may have corresponded to circular artifacts and the other around 0.79 mm^−1^ on UHR-SPCCT images, and around 0.26 mm^−1^ on HR–SPCCT images (Supplemental Fig. 2). For both images, the NPS peak intensities were higher for the low-frequency peak than for the other peak (Fig. 1). The same outcomes were found for conventional images with the second peak placed at higher spatial frequencies than those for spectral images for HR-SPCCT images and the opposite for UHR-SPCCT images.

### Task-based transfer function

**Fig. 2:**
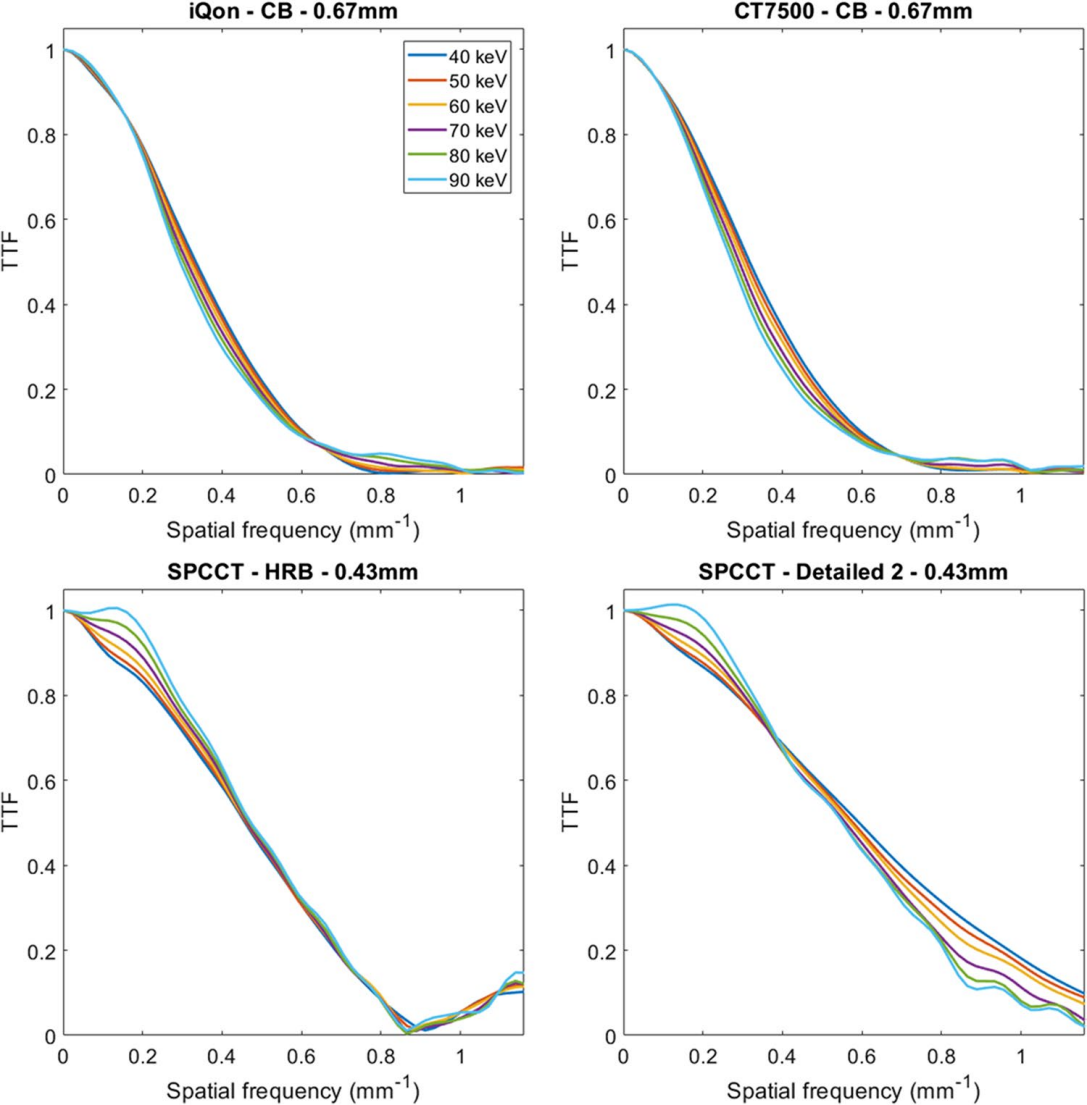
Task-based transfer function curves according to the energy levels obtained for dual-energy CT images from two dual-layer CT scanners with energy-integrating detectors (IQon CT, CT7500) and a spectral photon-counting CT (SPCCT). For SPCCT, raw data were reconstructed using high-resolution parameters with HRB kernel and 0.67-mm slice thickness, and ultra-high-resolution parameters with Detailed 2 kernel and 0.43 mm slice thickness

The task-based transfer function values of 50% (*f*_50_) for the iodine insert according to the energy levels (keV) for each CT system are depicted in Fig. 2.

For the CT7500 and IQon CT (Table 2), *f*_50_ values decreased from 40 to 90 keV (from 0.32 to 0.27 mm^−1^ and from 0.33 to 0.29 mm^−1^, respectively). The same pattern was found for UHR-SPCCT images (from 0.59 to 0.55 mm^−1^) while on the HR–SPCCT images, *f*_50_ values were similar from 40 to 90 keV (0.47 ± 0.05 mm^−1^).

For all keV, *f*_50_ values were lower with CT7500 than with IQon CT (− 6% ± 0.9%). For SPCCT, *f*_50_ values were higher on HR and UHR-SPCCT images than on HR-EID-DECT images. For all keVs, the use of UHR-SPCCT images increased *f*_50_ values compared to HR-SPCCT images with an average increase of 22% ± 4.9%. For SPCCT, *f*_50_ values were higher for conventional images than for spectral images at all energy levels.

### Contrast between the iodine insert and background material

**Fig. 3:**
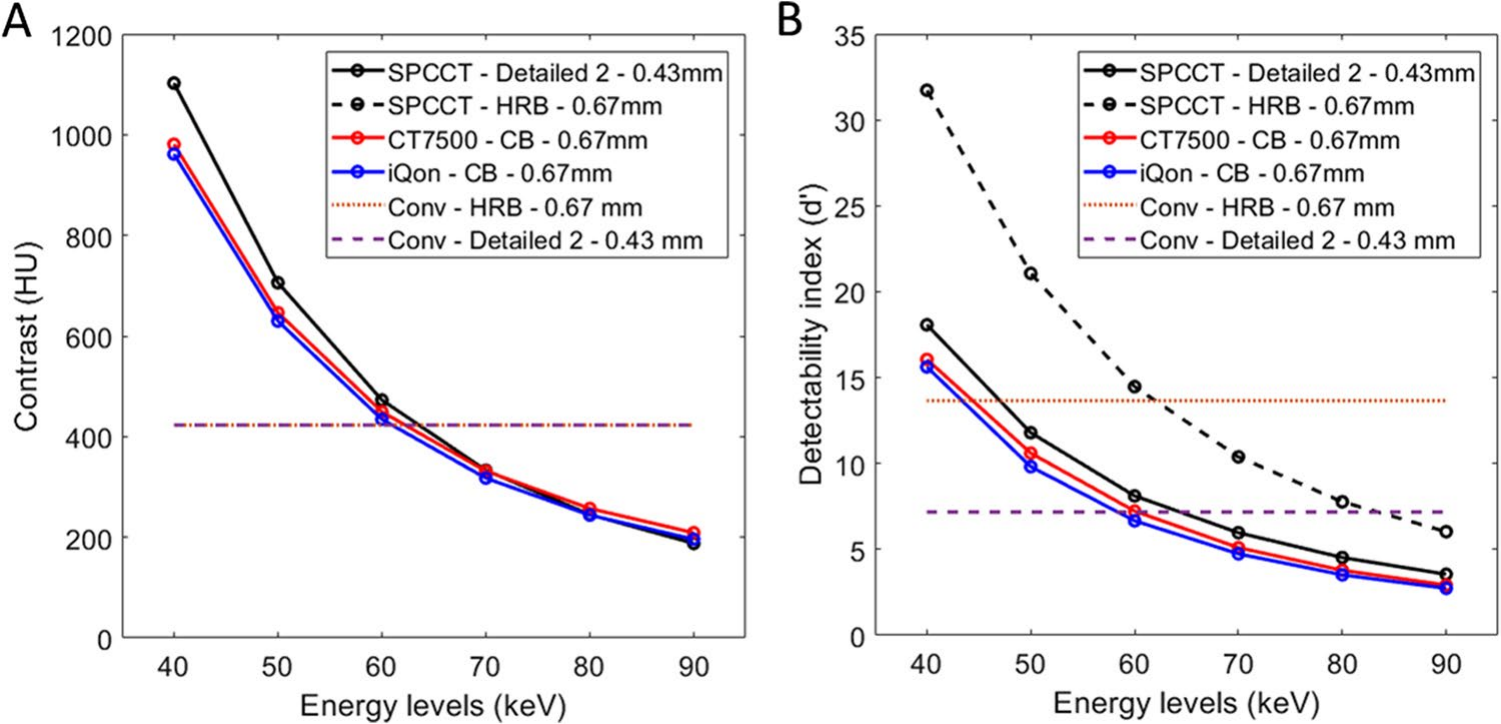
**A** Contrast between the phantom background material and the iodine insert according to the energy levels obtained for two dual-layer energy-integrating detectors dual-energy CT images (IQon CT, CT7500) and a spectral photon-counting CT (SPCCT). **B** Detectability index values of the simulated lesion according to the energy levels from 40 to 90 keV on the two DT platforms. For SPCCT, raw data were reconstructed using high-resolution parameters with HRB kernel and 0.67 mm slice thickness, and ultra-high-resolution parameters with Detailed 2 kernel and 0.43 mm slice thickness for conventional and spectral images

For all CT systems, the contrast between the iodine insert and background material decreased as the keV increased (Fig. 3A). For SPCCT, similar contrast values were found for both resolution images. They were higher with SPCCT than with the other CT systems from 40 to 70 keV but the opposite afterwards, and were slightly higher with CT7500 than with IQon (4% ± 1.7%).

### Detectability index

**Fig. 4:**
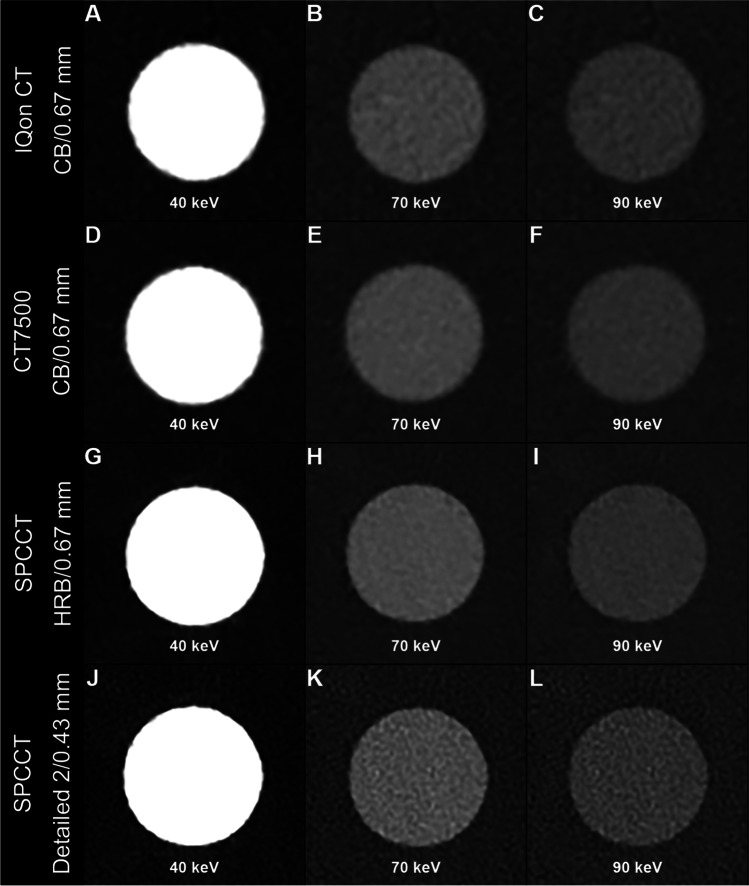
Virtual monochromatic images at 40 keV (**A**, **D**, **G**, **J**), 70 keV (**B**, **E**, **H**, **K**), and 90 keV (**G**, **F**, **I**, **L**) (WL: 150, WW: 1000) of the iodine insert used for task-based transfer function computation (Gammex Mercury 4.0^TM^ phantom) for dual-energy CT images from two dual-layer CT scanners with energy-integrating detectors (IQon CT, CT7500) and a spectral photon-counting CT (SPCCT). For SPCCT, raw data were reconstructed using high-resolution parameters with HRB kernel and 0.67 mm slice thickness, and ultra-high-resolution parameters with Detailed-2 kernel and 0.43 mm slice thickness

For all keVs, *d′* was higher with CT7500 than IQon (7% ± 2.1%) (Fig. 3B). *d′* was higher on UHR-SPCCT images than with IQon (24% ± 5.5%) or CT7500 CT (16% ± 4.3%). *d′* was higher on HR-SPCCT images than on UHR-SPCCT images (74% ± 4.0%). Case examples of VMIs on the iodine insert are provided in Fig. 4.

For SPCCT, *d′* values were higher with spectral images than with conventional images at energy levels equal to or lower than 60 keV.

### Patient analysis

Eight participants who underwent both coronary CTA on SPCCT and either IQon CT (*n* = 3) or CT 7500 (*n* = 5) were included (mean age: 61.5 years ± 12.7; mean body mass index: 26.1 ± 4.8 kg.m^2^; 2 women [25%]).

**Fig. 5:**
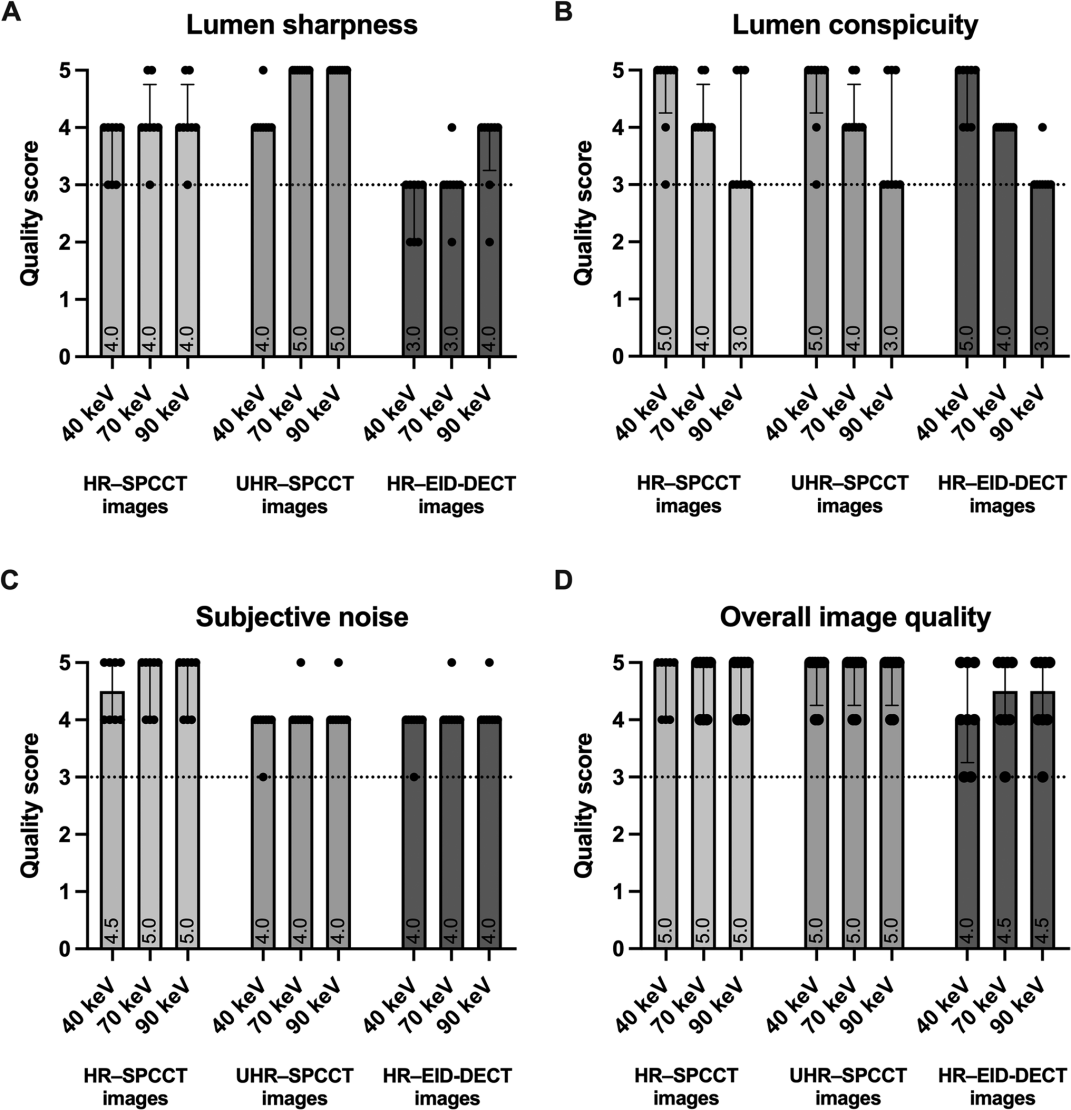
Scatter dot plots with bars of the subjective analysis in eight patients who underwent both a spectral photon-counting CT scan (SPCCT) and a dual-layer dual-energy CT scan with energy-integrating detectors (EID-DECT) (**A**: lumen sharpness, **B**: lumen conspicuity, **C**: subjective noise, **D**: overall image quality). Vertical bars show median values with interquartile range; black dots show score values; dotted horizontal line set at y = 3 show the minimal score acceptable for clinical use

**Fig. 6:**
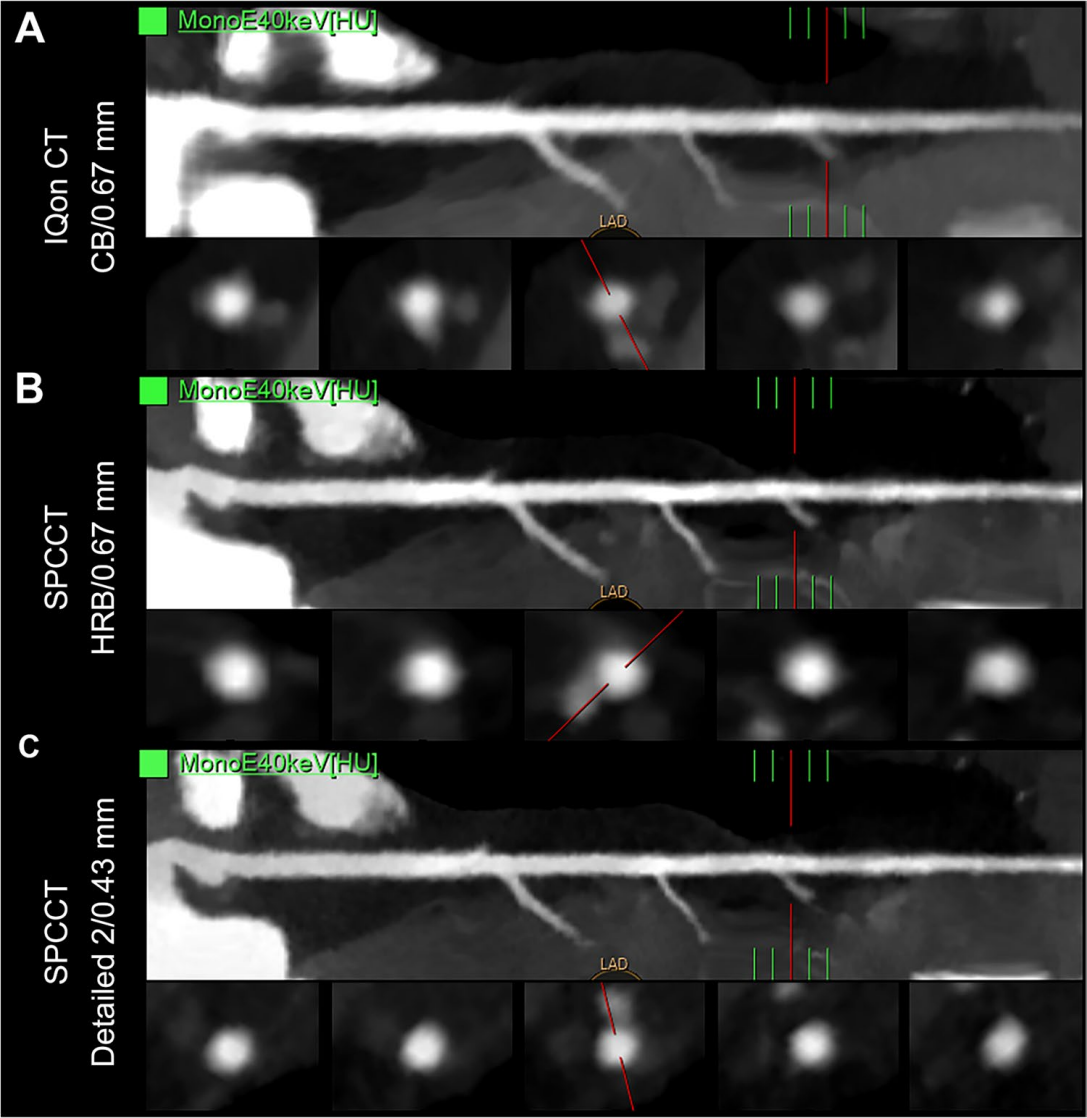
40 keV virtual monochromatic images (VMIs) curved planar reconstructions and cross axial images (WL: 1000, WW: 3000) of a left anterior descending coronary artery in a 44-year-old woman, who underwent both a dual-energy CT scan with energy-integrating detectors (EID-DECT: iQon CT) and a spectral photon-counting CT (SPCCT) (**A**: HR-EID-DECT, **B**: HR-SPCCT, **C**: UHR-SPCCT images). VMIs at 40 keV showed greater sharpness and conspicuity of the coronary lumina with SPCCT images for a greater overall image quality, compared with EID-DLCT images

**Fig. 7:**
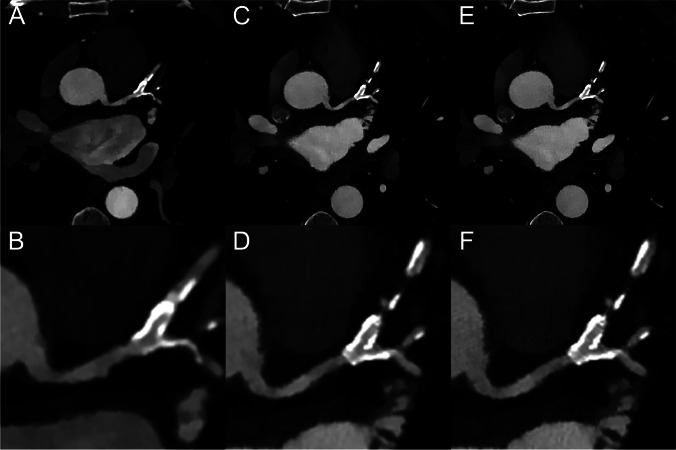
Example of a coronary CT angiography from an 8-cm collimation energy-integrating detector dual-energy CT scanner (EID-DECT, **A**, **B**: CB filter, 0.67 mm slice thickness) and a spectral photon-counting CT system (**C**, **D**: HRB filter, 0.67 mm slice thickness, **E**, **F**: Detailed 2 filter, 0.43 mm slice thickness) in a 69-year-old man. Top row: axial images at 40 keV (WL: 800, WW: 3000). Bottom row: magnified 40-keV axial images of the left anterior descending artery (WL: 800, WW: 3000). A greater lumen sharpness and conspicuity of the thoracic vessels are obtained with the SPCCT images, more particularly on the UHR-SPCCT images (**E**). On the magnified views, depiction of the coronary wall and the lumen, even in the presence of a stent, is improved on the SPCCT images (**C**–**F**) compared to the EID-DECT images (**A**, **B**)

Scores for subjective noise were almost similar on both CT images, whatever the energy level. They were good on the HR-EID-DECT images and UHR-SPCCT images whereas they were up to excellent on the HR-SPCCT images. Scores for lumen sharpness were greater on both SPCCT images compared to EID-DECT images for all energy levels (*p* < 0.001) whereas they were better on the UHR-SPCCT images than on the HR-SPCCT images (*p* < 0.001). Interestingly, scores for the UHR-SPCCT images proved to be excellent from 50 keV whereas neither HR-SPCCT nor HR-EID-DECT images were scored as excellent. It should be noted that a score below 3 was attributed to the HR-EID-DECT images for three patients between 40 and 50 keV. Scores for lumen conspicuity were excellent for all CT systems between 40 and 60 keV, good between 60 and 70 keV, and acceptable between 80 and 90 keV. Scores for overall image quality were greater on SPCCT images for all energy levels whereas they were slightly greater on UHR-SPCCT (Fig. 5 and Supplemental Table 1). In addition, the radiologists did not notice any circular artifacts like those seen on the phantom imaging (Figs. 6 and 7).

### Radiation dose

Radiation dose parameters between paired coronary CTA acquisitions were not significantly different. With SPCCT, the mean tube current (± SD) was of 302 ± 39 mAs, the CTDI_vol_ was 30.4 ± 3.9 mGy, and the DLP was 487 ± 63 mGy.cm. With EID-DECT, the mean tube current (± SD) was 387.5 ± 119 mAs, the CTDI_vol_ was 32.2 ± 11.1 mGy, and the DLP was 634 ± 195 mGy.cm.

## Discussion

Spectral CT technology is evolving quickly with the concomitant development of dual-energy and multi-energy spectral photon-counting CT (SPCCT) [2]. The latter, more recently introduced in the clinical field, holds great promise for overcoming the limitations of current energy-integrating detector CT devices such as dual-energy CT (EID-DECT) systems. In the present study, high-resolution (HR) and ultra-high-resolution (UHR) virtual monochromatic images (VMIs) for coronary artery imaging generated by SPCCT led to better-quality images than the HR images generated by EID-DECT. Using HR-SPCCT images, a task-based analysis showed a higher noise spatial frequency at comparable magnitude, higher spatial resolution, and higher detectability of the coronary lumen than with the HR-EID-DECT images. Using UHR-SPCCT images, the spatial resolution was much improved despite a higher noise and the detectability was maintained in comparison to the HR-EID-DECT images. Altogether, these results pave the way for potential improvements in spectral coronary CT angiography examinations using VMIs on SPPCT.

Using clinical routine HR parameters for cardiac imaging, image noise between CT systems was of similar magnitude with a slightly higher noise on IQon CT. However, compared to EID-DECT, the spatial frequency of HR-SPCCT changed dramatically with a strong shift in the NPS peak and TTF to higher frequencies, resulting in a higher spatial resolution and lower image smoothing. This effect was even more observable on the UHR images. Indeed, the Detailed-2 kernel conveys higher spatial resolution performances than the HRB kernel due to higher values of TTF at 20 and 50%. Also using VMIs with isotropic voxels at 0.43 mm^3^, the NPS peaks shifted towards high frequencies and were found for all energy levels. Altogether, this resulted visually in greater lumen sharpness on the phantom and the few patients studied. Furthermore, the noise showed similar performances for all VMIs generated with the three systems. This consistency may be explained by a similar approach in noise handling and anti-correlative noise that relies on the same detection-based spectral CT technology followed with projection domain spectral decomposition, such as previously reported using a 4-cm dual-layer EID-DECT [10, 42]. But it could also be explained by the electronic noise suppression made possible by the PCD which also benefits VMIs [42–45]. In addition, it is worth mentioning that a second NPS peak was observed at low frequencies on the SPCCT images. This may be related to residual band artifacts related to finite detector stability that might be further improved with more complex calibrations. This peak had a higher amplitude on UHR-SPCCT images which increased with lower-keV VMIs. But these artifacts were not noticed in the patient study, which suggests a dependence on the shape and composition of the phantom, as shown in a previous study [36].

As a result of the NPS and TTF performances, the task-based analysis showed higher detectability indexes (*d′*) for a simulated 2-mm lesion on HR-SPCCT images than on HR-EID-DECT images. Secondly, the *d′* was lower on UHR-SPCCT images than on HR-SPCCT images due to higher image noise, despite better noise texture and spatial resolution. Nevertheless, it was slightly better compared to the HR-EID-DECT images showing that UHR parameters provided more gain than the loss due to higher noise levels. Third, *d′* increased at a lower energy level mainly due to the photoelectric effect which is predominant at low energy particularly close to the K-edge of iodine, around 30.3 keV. Altogether, the radiologist confirmed these findings by showing that the overall image quality with SPCCT images had improved. A greater difference between SPCCT and EID-DECT images was particularly reported at low VMIs, which supports a higher gain in quality than for high VMIs. These findings are particularly interesting as they may allow greater performance in coronary stenosis quantification than what was previously reported with EID-DECT systems [46]. In addition, it is worth mentioning that, using HR-EID-DECT images between 40 and 50 keV, sharpness was scored as suboptimal for one third of patients whereas, with SPCCT images, the quality of sharpness was always more than satisfactory, with a higher score on the UHR-SPCCT images. Finally, the *d'* values obtained at 40, 50, and 60 keV were higher than those obtained for conventional acquisitions for both kernels with the SPCCT. Consequently, these results show the strong interest of low-keV VMIs for improving detection and quantification of the coronary lumen.

Additional interesting results have been shown in the phantom study such as an increase in CT numbers on VMIs with SPCCT, compared to VMIs with EID-DECT systems. This may be explained by a constant weight of incoming photons provided by the PCD that enable higher weight of the low-energy photons that carry the photoelectric effect, compared to the linear weight performed by the EID [1, 13]. This finding is expected with SPCCT technology and partly explains its ability to better detect high-contrast lesions such as coronary calcifications or pulmonary nodules [17, 18, 47–49]. Second, neither HR-SPCCT nor UHR-SPCCT images showed any evidence of a strong loss in spatial resolution at low energy levels, in comparison with HR-EID-DECT. This emphasizes better mitigation of spatial resolution loss at low energy levels with SPCCT and may be explained by its improved sampling of transmitted spectrum energies. This enables better discrimination between low- and high-energy photons compared to the EID-DECT technology and opens the way towards better resolution of photoelectric and Compton effects that can be combined with UHR capabilities, explaining the strength of this new technology [15].

Our study has limitations. First, we did not investigate the impact of the 1024 matrix size for SPCCT which may offer greater spatial resolution performances. Second, the phantom was static, which may lead to an overestimation of the detectability performance. However, at similar gantry revolution time, the overestimation magnitude was the same for both systems; noise and spatial resolution properties are given by the systems’ design. Third, we did not evaluate the impact of the iDose^4^ levels for SPCCT and of the spectral levels for dual-layer EID-DLCT. Fourth, the human study was limited to only eight patients and did not investigate the diagnostic performances of stenosis evaluation. Only high-contrast coronary arteries were studied, and further investigations are required to determine whether this could be applied to low-contrast areas such as the myocardium. Finally, and more importantly, the outcomes of the patient study are neither a clinical study nor a validation of the phantom results, but an in vivo clinical illustration. Our outcomes must now be validated for routine coronary SPCCT angiography by a large cohort of patients.

In conclusion, the quality of virtual monochromatic images for coronary artery imaging from 40 to 90 keV of a spectral photon-counting CT surpassed the quality of images from two dual-energy CT scanners, from the same manufacturer, with dual-layer energy-integrating detectors. SPCCT showed an improvement in spatial resolution, noise magnitude and texture for better detectability of the coronary lumen using high-resolution and ultra-high-resolution parameters.

### Supplementary Information

Below is the link to the electronic supplementary material.

Supplementary file1 (DOCX 23406 KB)

## Abbreviations

CTDI_vol_: Volume CT dose index
EID-DECT: Energy-integrating detectors–dual-energy computed tomography
ESF: Edge-spread function
HR: High resolution
iDose^4^: Intelligent dose
IR: Iterative reconstruction
LSF: Line-spread function
NPS: Noise power spectrum
PCD: Photon-counting detector
SPCCT: Spectral photon-counting computed tomography
TTF: Task-based transfer function
UHR: Ultra-high resolution
VMI: Virtual monochromatic image

## Declarations

### Guarantor

The scientific guarantor of this publication is Pr Philippe Douek.

### Statistics and biometry

No complex statistical methods were necessary for this manuscript.

### Study subjects or cohorts overlap

Two out of 8 subjects have been previously reported in a previous paper. But the scope of this study was not the image quality of virtual monochromatic images.

### Methodology

• Prospective for the patient study

• Experimental for the phantom study

• Performed at one institution
