# Supplementary material for: Virtual monochromatic images for coronary artery imaging with a spectral photon-counting CT in comparison to dual-layer CT systems: a phantom and a preliminary human study
Source: Eur Radiol. 2023 Mar 15;33(8):5476–88. doi: 10.1007/s00330-023-09529-9 (PMC10326132; doi:10.1007/s00330-023-09529-9)

**Supplemental Table 1.** Subjective analysis in eight patients who underwent coronary CTA with both a dual-layer energy-integrating detectors dual-energy CT (IQon CT or CT7500) and a spectral photon-counting CT (SPCCT). Image interpretation was performed in consensus by two senior cardiac radiologists. They assessed the subjective noise, the lumen conspicuity and sharpness and the overall image quality using a commonly used five-point Likert scale (1 = unacceptable, 2 = suboptimal, 3 = acceptable, 4 = above average, and 5 = excellent). A value of less than 3 was considered unsatisfactory for clinical use.

| **Virtual monochromatic images** | | **40 keV** | **50 keV** | **60 keV** | **70 keV** | **80 keV** | **90 keV** |
| --- | --- | --- | --- | --- | --- | --- | --- |
| **Lumen sharpness** | HR–SPCCT images | 4 [3; 4] | 4 [3.25; 4] | 4 [4; 4.75] | 4 [4; 4.75] | 4 [4; 4.75] | 4 [4; 4.75] |
|  | UHR–SPCCT images | 4 [4; 4] | 5 [4.25; 5] | 5 [5; 5] | 5 [5; 5] | 5 [5; 5] | 5 [5; 5] |
|  | HR–EID-DECT images | 3 [2; 3] | 4 [2; 3] | 3 [3; 3] | 3 [3; 3] | 3.25 [4; 4] | 4 [3.25; 4] |
| **Lumen conspicuity** | HR–SPCCT images | 5 [4.25;5] | 5 [4.25;5] | 4 [4; 4.75] | 4 [4; 4.75] | 3 [3; 3.75] | 3 [3; 3.75] |
|  | UHR–SPCCT images | 5 [4.25; 5] | 5 [4.25; 5] | 4 [4; 4.75] | 4 [4; 4.75] | 3 [3; 4.75] | 3 [3; 3] |
|  | HR–EID-DECT images | 5 [4; 5] | 5 [4; 5] | 4 [4; 4.75] | 4 [4; 4] | 3 [3; 3] | 3 [3; 3] |
| **Subjective noise** | HR–SPCCT images | 4.5 [5;5] | 5 [4;5] | 5 [5;5] | 5 [5;5] | 5 [4;5] | 5 [4;5] |
|  | UHR–SPCCT images | 4 [4; 4] | 4 [4; 4] | 4 [4; 4] | 4 [4; 4] | 4 [4; 4] | 4 [4; 4] |
|  | HR–EID-DECT images | 4 [4; 4] | 4 [4; 4] | 4 [4; 4] | 4 [4; 4] | 4 [4; 4] | 4 [4; 4] |
| **Overall image quality** | HR–SPCCT images | 5 [4; 5] | 5 [4; 5] | 5 [4; 5] | 5 [4; 5] | 5 [4; 5] | 5 [4; 5] |
|  | UHR–SPCCT images | 5 [4.25; 5] | 5 [4.25; 5] | 5 [4.25; 5] | 5 [4.25; 5] | 5 [4.25; 5] | 5 [4.25; 5] |
|  | HR–EID-DECT images | 4.5 [3.25; 5] | 4.5 [3.25; 5] | 4.5 [4; 5] | 4.5 [4; 5] | 4.5 [4; 5] | 4.5 [4; 5] |

*Footnote– HRB (high-resolution standard filter) and CB (cardiac standard filter) were used for high-resolution (HR) imaging in combination to 0.67 mm slice thickness. Detailed 2 was used for ultra-high-resolution (UHR) imaging in combination with 0.43 mm slice thickness. SPCCT: spectral photon-counting CT, EID-DECT: energy integrating detectors dual-energy CT.*

*Data are median [1^st^ quartile – 3^rd^ quartile].*

## SUPPLEMENTAL FIGURE CAPTIONS

**Supplemental Figure 1.** Task-based image quality assessment on HR–SPCCT images.

1. Four regions of interest used for the noise power spectrum (NPS) assessment.
2. Region of interest (in blue) used to compute the task-based transfer function (TTF) with the iodine insert at 10 mg/mL.


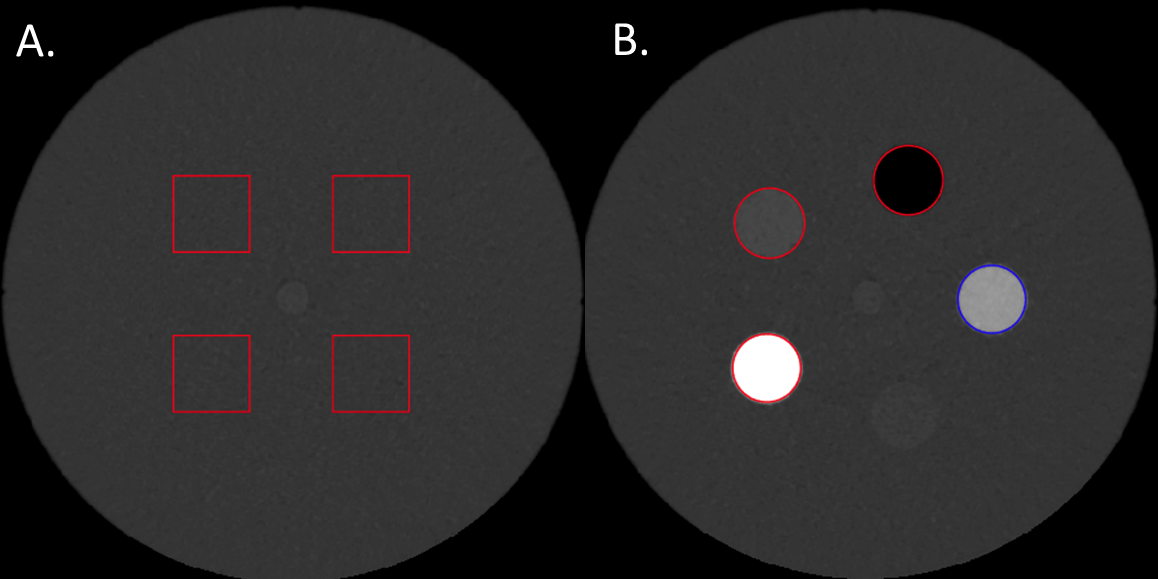


**Supplemental Figure 2.** Virtual monochromatic images of a coronary spectral photon-counting CT (SPCCT) protocol on the homogeneous module of Mercury 4.0 phantom (WL: 600, WW: 4000, A: 70 keV images with HR–SPCCT images, B: 40 keV images with the HR–SPCCT images, C: 70 keV images with the UHR–SPCCT images, D: 40 keV images with the UHR–SPCCT images). Axial images show circular artifacts measured as the lowest peak frequency in the Noise Power Spectrum (NPS) analysis. Despite their presence, both magnitude and the average frequency of the NPS were much better on the SPCCT images than on the dual-energy CT scans with energy-integrating detectors.


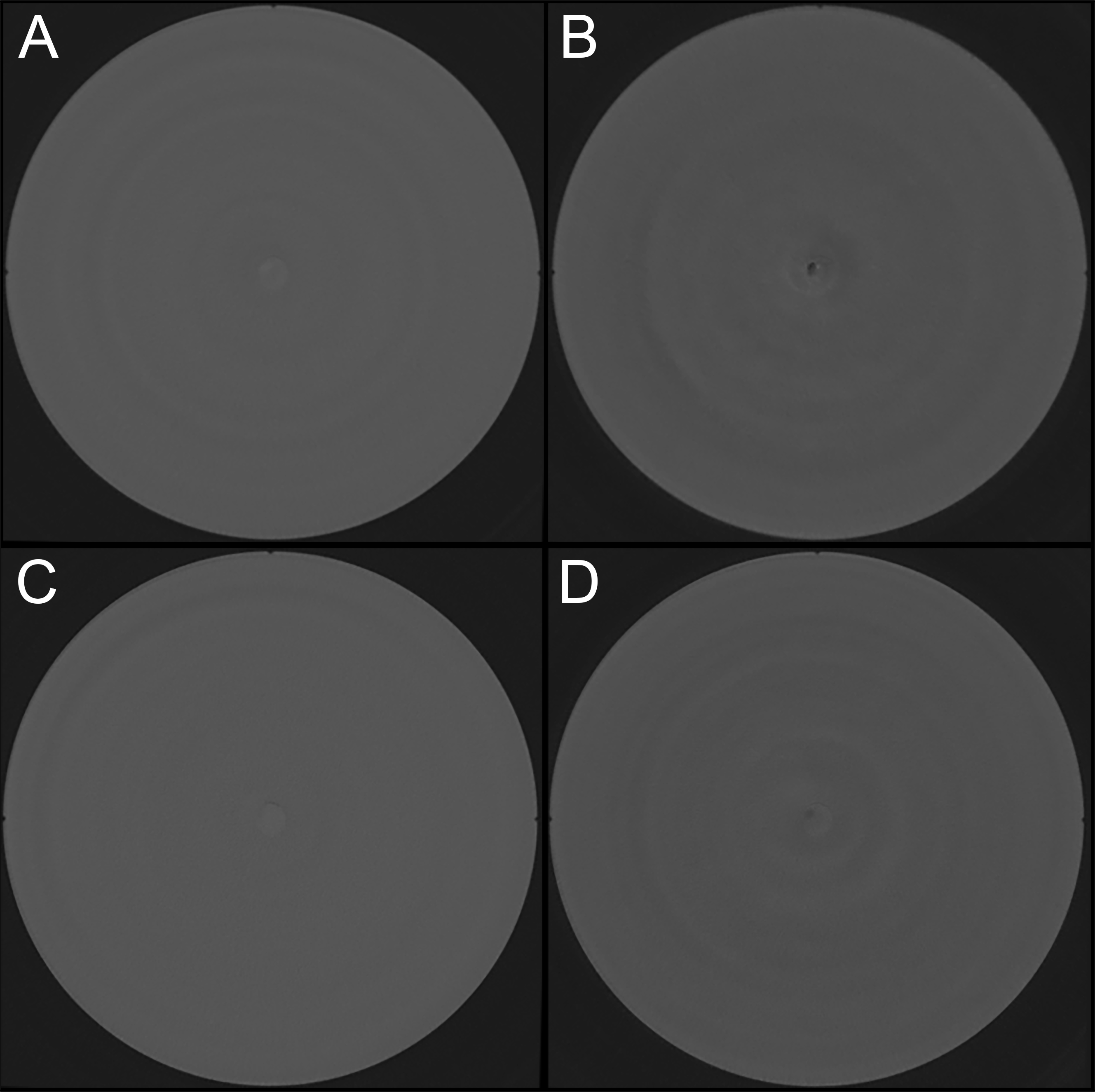

Supplement: Supplementary file 1 — Supplementary file1 (DOCX 23406 KB) [file 330_2023_9529_MOESM1_ESM.docx]
